# Sex Differences in Attentional Selection Following Gaze and Arrow Cues

**DOI:** 10.3389/fpsyg.2020.00095

**Published:** 2020-02-14

**Authors:** Jeanette A. Chacón-Candia, Juan Lupiáñez, Maria Casagrande, Andrea Marotta

**Affiliations:** ^1^Dipartimento di Psicologia, Sapienza Universitá di Roma, Rome, Italy; ^2^Department of Experimental Psychology and Mind, Brain, and Behavior Research Center (CIMCYC), University of Granada, Granada, Spain; ^3^Dipartimento di Psicologia Dinamica e Clinica, Sapienza Universitá di Roma, Rome, Italy

**Keywords:** attentional selection, gaze-cueing, theory of mind, autistic quotient, sex differences

## Abstract

Although most studies on social attention have shown undistinguishable attentional effects in response to eye-gaze and arrow cues, recent research has found that whereas the orienting of attention triggered by eye-gaze is directed to the specific position, or part of the object looked at, arrows unselectively elicit attention toward parts of the environment. However, it is unclear whether this dissociation between gaze and arrow cues is related to social cognitive mechanisms such as mental state attribution (Theory of Mind, ToM). We aimed at replicating the dissociation between gaze and arrow cues and investigating if the attentional object selection elicited by these two types of stimuli differs depending on the sex of observers. To make our research plan transparent, our hypotheses, together with the plans of analyses, were registered before data exploration. While we replicated the arrow–gaze dissociation, this was equivalent in the male and female population. These results seem to contradict the intuition that ToM skills can be associated with the differences observed between orienting to eyes and arrows since greater ToM abilities have been generally shown in females. However, this conclusion must be interpreted with caution, since, in our sample, it was not possible to observe any differences in autistic quotient scores and ToM abilities between male and female participants. Further research is needed in order to clarify this issue.

## Introduction

Past research has suggested that females generally outperform males on various tests of social abilities, such as cognitive and emotional perspective-taking, empathy, eye-contact, emotional expression detection, and “mindreading” abilities (Bosacki, 2000; Suzuki et al., 2006; Voracek and Dressler, 2006; Alwall et al., 2010; Derntl et al., 2010). Sparse research available concerning gender differences in selective attention thus far suggests that this may be an important component of cognitive gender differences (Bayliss et al., 2005). The question we address in the current study is whether males and females differ in the attentional object selection elicited by eye-gaze direction.

The tendency to direct our attention to where other individuals are looking at has been the centre of interest of a large number of studies (Birmingham and Kingstone, 2009; Nummenmaa and Calder, 2009). This behavior appears from an early age (Batki et al., 2000; Hood et al., 1998) and represents a crucial step to develop social communication, since gaze offers several pieces of information about action goals, feelings, and beliefs of another person (Emery, 2000).

Such findings imply that the perceptual and attentional systems preferentially process eye-gaze direction and this preference has been generally considered to reflect the central role of gaze signals in the development of communicative competences including cultural acquisition, language learning, and mental state attribution (Baron-Cohen,1995; Tomasello, 1995), with atypical developmental patterns frequently associated with social dysfunctions, such as autism (e.g. Baron-Cohen, 1995; Swettenham et al., 1998). Given this, it is not surprising that some authors have suggested that eye-gaze cues are unique to shift attention (e.g. Farroni et al., 2002).

Thus, several studies have tried to distinguish between the attentional orienting triggered by social stimuli like gaze and non-social cues such as arrows employing the traditional gaze-cueing paradigm (Friesen and Kingstone, 1998), showing no robust behavioral differences between arrow and gaze cues (see, Ristic et al., 2002; Tipples, 2008; Birmingham and Kingstone, 2009; Galfano et al., 2012). However, in recent years, the uniqueness of the eye-gaze for the human attentional system continues to be demonstrated in a growing number of investigations through distinct methodologies.

For example, using a visual memory task, Dodd et al. (2012) and Gregory and Jackson (2017) have studied the difference between gaze and arrow cues, showing an improvement in memory accuracy just when information is cued by a gaze but not when using an arrow. Moreover, Marotta et al. (2018) observed that eye-gaze and arrows yielded opposite spatial interference effects when used as targets in a spatial interference task: whereas arrows elicited the usual spatial stroop effect, i.e. faster reaction times when its position was congruent with the direction, eye-gaze produced the opposite effect, i.e. faster responses when it was incongruent. Another stream of studies showed dissociations between gaze and arrows within clinical populations, such as schizophrenia, or ADHD (e.g. Dalmaso et al., 2013; Marotta et al., 2014, 2017).

Relevantly, research by Marotta et al. (2012) have also shown different forms of attentional selection between eye-gaze and arrows even with a gaze-cueing paradigm. Authors displayed two rectangles, in which one end or another of one of them was cued by a central non-informative directional eye-gaze or arrow cue, and then succeeded by a target presented in one end of those rectangles. It was found that arrows triggered attentional orienting that spread to the entire object (i.e. even to the other end of the rectangle), whereas gaze triggered attentional orienting exclusively to the rectangle end specifically looked at. On the basis of these results, the authors proposed that whereas arrow-cueing is truly stimulus-driven, the attentional orienting to eye-gaze may be mediated by mental state attribution. In particular, according to Marotta et al.’s (2012) view, “The specific location-based effect observed with eye-gaze cues seems consistent with the idea that gaze reflects ‘social’ processing and that an intention is attributed to the gaze to look at a specific location. […] Hence, we jointly orient our attention specifically to the inferred location within the object of interest, not to the entire object. In contrast, the object-based effect of arrow cues may be triggered by a more unspecified directional code that automatically orients attention through the entire placeholder object”(p. 333).

However, it is important to note that the study of Marotta et al. (2012) was the first that ever assessed the type of attentional selection elicited by eye-gaze and arrow cues and that most of the participants of the study were female. For this reason, assuming the natural variations in the effect triggered by gaze cues across individuals, the interpretation of the findings observed in their study must be cautious and should not be necessarily extended to the general population. Indeed, some individuals could be oriented strongly toward social stimuli, while others may not. Some studies, for example, have shown, that the gaze-cueing effect is weaker in individuals reporting autistic-like traits (Bayliss et al., 2005; Alwall et al., 2010) and more robust in observers with low self-esteem (Wilkowski et al., 2009).

Importantly, Bayliss et al. (2005) observed that the sex of participants also counts as part of the individual differences found in the gaze-cueing effect. In particular, they reported that females had a stronger gaze-cueing effect than male participants and that there was a negative correlation between cueing effects and Autism Spectrum Quotient scores (AQ; Baron-Cohen et al., 2001). Thus, they speculate that, across gender, people who have more social skills tend to show a larger gaze-cueing effect.

Based on the natural variations of the gaze attentional effect across individuals and the gender differences observed in the studies mentioned above, the aims of the present study were the following:

We firstly tried to replicate Marotta et al.’s (2012) dissociation between gaze and arrow attentional orienting: attention will be directed to the entire object (not only the indicated end of the rectangle) with arrow cues, while it will selectively be oriented to the specific position or part of the object where eye-gaze cues are looking at.

Secondly, we investigated if this dissociation is only observed in female participants or it can be generalized regardless of sex. Since it has been generally observed that females outperform males in social abilities and cognition (Bosacki, 2000; Suzuki et al., 2006; Voracek and Dressler, 2006; Alwall et al., 2010; Derntl et al., 2010), we expect that the dissociation between gaze and arrows will be particularly evident in female participants.

Thirdly, we looked for associations between this dissociation, autistic traits (as measured by the AQ; Baron-Cohen et al., 2001) and theory of mind skills (as measured by the Yoni Task, Shamay-Tsoory and Aharon-Peretz, 2007), the hypothesis being that people with more autistic traits and/or low theory of mind would not show a dissociation between gaze and arrow cues.

The hypotheses for this experiment, together with the plans of analyses, were registered before data exploration in Open Science Framework^1^.

## Materials and Methods

### Participants

Fifty-two university students provided their informed consent before voluntarily participating in this study; 26 males (mean age = 21.73), and 26 females (mean age = 20.03). All of them had normal or corrected-to-normal vision and were naïve about the purpose of the research. A minimum of 24 participants per group (24 men and 24 women) was intended as in the original study by Marotta et al. (2012). Although no power analysis was performed *a priori*, a sensitivity analysis using G^∗^power (Faul et al., 2007), showed that with our final sample size (*N* = 52), the minimum effect size that could have been detected for α = 0.5, and 1 −β = 0.95, for 2 groups and 4 within-participants conditions (for each of the critical CT relation × Type of Cue analyses) is *f* = 0.40 (minimum detectable effect).

### Measures

#### Double Rectangle Task

**FIGURE 1:**
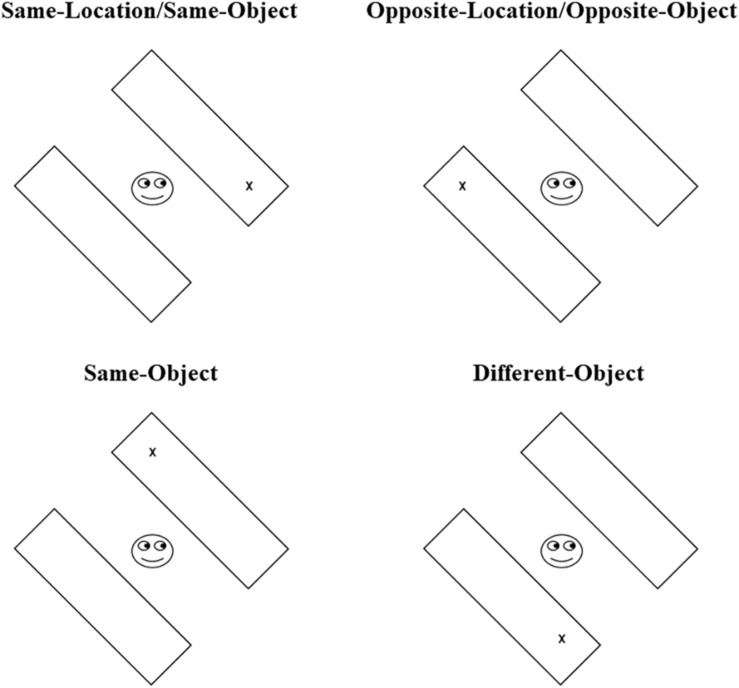
Illustration of the four cue-target (CT) relation conditions. The display orientation depicted here is −45° from vertical (Marotta et al., 2012).

The double rectangle task used in this study was very similar to the one used by Marotta et al. (2012) in their experiments 1 and 2, although some changes were made to the procedure. More specifically, both the rectangle orientation (+ 45° or −45° tilted from the vertical meridian; see, Figure 1) and the type of cue (arrows and eye-gaze) were randomly interspersed in each of the three experimental blocks of trials, whereas one of these variables was blocked in the original study. These changes were made to ensure that differences between eye-gaze and arrows are due to different selection mechanisms rather than to different between-block strategies. Each trial began with a central fixation stimulus and two rectangular objects (subtending 10.5° × 3° of visual angle) that appeared in one of the two possible orientations. The fixation stimuli changed depending on the cue type. As in the Marotta et al. (2012) study, in gaze-cueing trials, the fixation was a central schematic happy face^2^ (3° × 2.5°) with the pupils straight, whereas, in arrow trials, the fixation was a central cross (0.5° × 2°). This display was presented for 700 ms; then, a change was made either to the arrow or eye-gaze cue to indicate one end of the two rectangular objects. The target followed after 150, 300, or 600 ms in one of four rectangles end according to the four critical cueing conditions (see, Figure 1): at the cued direction (and object) indicated by the cue (same-location/same-object trials), in the opposite object and direction to which the cue was directed (opposite-location/opposite-object trials), at the uncued location of the same object (same-object trials), or at the uncued location in the other object (different-object trials). Participants were asked to respond promptly to target stimuli (the letter “X” or “O”) by pressing either the “C” key (with the left hand) or the “M” key (with the right hand) on the computer keyboard, depending on the presented target letter. Half of the participants pressed “C” when the letter “X” appeared as a target and “M” when the letter “O” appeared, whereas the other half received the reverse mapping. This task consisted of four blocks of trials; one of them was a practice block with just 10 trials; the other three were the experimental blocks with 192 trials each, summing up 576 experimental trials in total, with 72 observations per experimental condition. Target location, cue direction, type of cue, and the object orientation were randomized within each block of trials.

#### Yoni Task

The “Yoni task” (Shamay-Tsoory and Aharon-Peretz, 2007) is a computerized task inspired by Baron-Cohen (1995), which measures the ability to attribute mental states based on the eye direction of a cartoon face (“Yoni”) and verbal cues. This task is designed to separately assess cognitive and affective ToM. In the cognitive conditions, the verbal cue and Yoni’s facial expression are emotionally neutral, and in the affective one, those same cues offer affective information. In each trial, Yoni’s face surrounded by four colored objects or faces is presented in the middle of the screen and an incomplete sentence is presented at the top of the screen. Participants are required to read the sentence and click the cursor, using a mouse, on the image that they believe Yoni is referring to. The cognitive and affective conditions require either a first- or a second-order inference. In the first-order ToM conditions, participants were required to infer the mental state of “Yoni.” In the second-order ToM condition, participants were asked to understand Yoni beliefs about others’ beliefs and desires.

#### The Autism-Spectrum Quotient (AQ)

The AQ is a 50-item self-report questionnaire designed for measuring autistic traits in the general population (Baron-Cohen et al., 2001). In particular, it assesses five different domains relevant for autistic traits: social skills, attention to detail, attention switching, communication, and imagination. A Spanish version^3^ of this instrument has been used specifically for quantifying where participants are situated on the continuum from autism to normality.

### General Procedure

All participants were first required to perform both the Double Rectangle and the Yoni tasks; then, the AQ questionnaire was administered. The order of tasks was counterbalanced across participants. The study was conducted in accordance with the ethical standards of Declaration of Helsinki and was approved by the Ethical Committee of the University of Granada (175/CEIH/2017). All participants gave written informed consent before testing.

### Data Analysis

Given the specific hypotheses for the Double Rectangle task, separate two-factor repeated measures designs were used in order to analyze “general cueing” and “object-based cueing” effects, respectively, for targets appearing at the right and left locations, and for targets appearing at the bottom and top locations. Cue–Target (CT) relation consisted of four trial types: same-location/same-object trials and opposite-location/opposite-object trials, for the analysis of the general cueing effect; same-object trials and different-object trials for the object-based cueing effect. As in the Marotta et al. (2012) study, and anticipating irrelevant differences between vertical and horizontal target locations, this approach was taken because opposite-location/opposite-object trials were always paired with a horizontal target, whereas same-object/different-object trials were always paired with a vertical target. Type of Cue had two levels: eye-gaze and arrow^4^. Sex also had two levels: male and female. Planned comparisons were used for the analysis of interactions.

To analyze participants’ social abilities, one-way analyses of variance (ANOVAs) considering SEX (male/female) as an independent variable were performed both on the AQ score and on the Yoni test cognitive and affective accuracy scores. Data from one of the participants were excluded from the analysis of the Yoni test due to a technical error. Finally, to test the associations between cueing effects and autistic traits and ToM skills, Pearson correlations were calculated. Pearson correlations were also calculated between the index of the arrow/gaze object dissociation (measured as a difference between the object-cueing effect for arrow and the object-cueing effect for gaze cues) and autistic traits and ToM skills.

To get additional support for the obtained effects, we also computed their Bayes factors. By convention, when Bayes factor is above the value of 3, it can be taken as substantial evidence for the tested hypothesis, whereas when values are less than 1/3, these should be considered as substantial evidence for the contrasting hypothesis (Lee and Wagenmakers, 2014).

## Results

### Double Rectangle Task

Mean response times, standard deviations, and error percentages are presented in Table 1. RTs faster than 100 ms or slower than 1000 ms (0.2%) and incorrect response trials (4%) were excluded from the RT analysis in all conditions.

**TABLE 1:** Mean Reaction times (RT), Incorrect Rate (IR%), and Standard Deviation (SD) as a function of Sex, Type of Cue, and CT relation on the General-Cueing effect (same-location/same-object trials[SamLoc] and opposite-location/opposite-object trials[OppLoc]) and the Object-Based effect (same-object trials[SamObj] and different-object trials[DifObj]).

	General cueing effect							Object based effect							
		
	Gaze				Arrow			Gaze				Arrow		
				
	CT relation	RT	SD	IR(%)	RT	SD	IR(%)	CT relation	RT	SD	IR(%)	RT	SD	IR(%)
Male Group	SamLoc	491.0	46.55	4.5%	491.4	44.52	4.4%	SamObj	500.2	47.97	3.8%	503.9	50.49	4.5%
	OppLoc	499.2	52.06	3.6%	506.7	51.42	4.5%	DifObj	496.4	50.23	3.3%	510.4	48.75	4.5%
Female Group	SamLoc	509.9	52.90	4.5%	503.2	49.50	4.5%	SamObj	519.3	47.97	2.8%	520.2	52.76	3.3%
	OppLoc	524.6	52.71	4.0%	520.8	56.20	3.4%	DifObj	519.3	48.23	3.5%	529.6	50.63	4.0%

#### General-Cueing Effect

**FIGURE 2:**
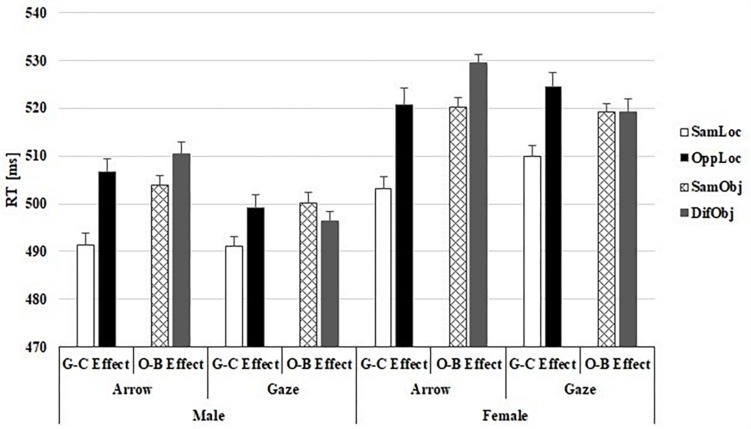
Reaction times (RT) results shown separately for male and female. Mean RTs presented for each cue type condition (gaze and arrow) as a function of cue-target relation (CT) in the General-Cueing effect (G-C Effect) and the Object-Based effect (O-B Effect). Error bars represent the standard error of the mean for each condition, computed following Cousineau’s (2005) method to eliminate variability between participants.

The ANOVA revealed a main effect of CT relation (*F*_1_,_50_ = 39.22; *p* < 0.001, η^2^_*p*_ = 0.44), showing that RTs were faster on same-location/same-object trials (*M* = 499 ms) than in opposite-location/opposite-object trials (*M* = 513 ms). Importantly, the CT relation × Type of Cue interaction was not significant (*F*_1_,_50_ = 1.15). The Sex × Type of Cue interaction was significant (*F*_1_,_50_ = 6.48; *p* = 0.014, η^2^_*p*_ = 0.11): female participants showed slower RTs for gaze than arrow cues (*F*_1_,_50_ = 4.26; *p* = 0.044), while male participants showed no differences in overall RTs between the two types of cues (*F*_1_,_50_ = 2.36; *p* = 0.131). However, neither the interaction Sex × CT relation (*F* < 1), nor the Sex × Cue Type × CT relation interaction (*F* < 1; Figure 2) was significant.

Bayes factor analyses were conducted to seek evidence in favor of the alternative hypothesis by contrasting models containing the effect to equivalent model stripped of the effect of interest. These analysis revealed at least anecdotal evidence in favor of the null hypothesis for Sex × Cue (BF_10_ = 0.88), Sex × CT relation (BF_10_ = 0.35), and Sex × Cue × CT relation (BF_10_ = 0.21).

The analyses of error rate expose a significant effect of Cue Type relation (*F*_1_,_50_ = 4.24; *p* = 0.045, η^2^_*p*_ = 0.07), showing that participants made more errors on valid (4.5%) than on invalid trials (3.9%). No other effects were significant.

#### Object-Based Effect

The ANOVA showed a main effect of the Cue Type (*F*_1_,_50_ = 12.68; *p* < 0.001, η^2^_*p*_ = 0.20), with longer RTs for the arrow cue (*M* = 516 ms) than for the gaze cue (*M* = 509 ms) condition. The main effect of the CT relation was also significant (*F*_1_,_50_ = 4.07; *p* = 0.049, η^2^_*p*_ = 0.07). Importantly, the CT relation × Cue Type interaction was significant (*F*_1_,_50_ = 10.49; *p* = 0.002, η^2^_*p*_ = 0.17). As can be observed in Figure 2, RTs were faster on same-object trials than on different-object trials, when using arrows as cues (*F*_1_,_50_ = 14.59; *p* ≤ 0.001, η^2^_*p*_ = 0.22). However, when eye-gaze was used, no differences were evident between same-object and different-object trials (*F* < 1). Finally, neither the Sex × CT relation interaction nor the Sex × Cue Type × CT relation interaction was significant (all *F*s < 1; see, Figure 2). Again, Bayes factor analyses showed moderate evidence supporting the null hypothesis for Sex × Cue (BF_10_ = 0.31), Sex × CT relation (BF_10_ = 0.22) and Sex × Cue × CT relation (BF_10_ = 0.25) interactions.

In the analyses of error rate, only the main effect of Cue Type approached significance (*F*_1_,_50_ = 3.99; *p* = 0.051, η^2^_*p*_ = 0.07), indicating that participants made slightly more errors on the arrow (4%) than on the gaze condition (3.3%). No other effect or interaction was significant.

### Sex Differences in Social Skills

AQ scores and ToM accuracy (as measured by the Yoni Task) were not different in the two groups of participants. Mean (± SD) and *t*-test results are reported in Table 2. Note that there were no Sex differences for any of the measured variables [AQ, female range 17 (6–23), male range 17 (7–24); ToM accuracy female range 0.33 (0.66–1), male range 0.33 (0.66–1)]. Correspondent Bayes factor analyses were also computed to assess how much support we have for the alternative hypothesis, specifying that females have higher social skills than men. As can be observed in Table 2, most BF_10_ were below 1 or close to it.

**TABLE 2:** Means, standard deviations (SD), and *t*-test results to assess differences between male and female participants on social skills considering AQ scores and Yoni test accuracy.

	Male		Female				
		
	Mean	SD	Mean	SD	*t*	*P*	BF_10_
AQ_Total	13.46	4.38	13.84	4.68	0.306	0.761	0.35
Affective 1	96.40	8.30	98.10	8.30	0.721	0.474	0.51
Cognitive 1	94.60	11.10	97.80	4.40	1.353	0.182	1.05
Affective 2	87.30	11.80	86.00	13.3	–0.352	0.726	0.22
Cognitive 2	82.90	13.10	75.40	18.4	–1.665	0.102	0.12

#### Correlations

To test the associations between cueing effects on the one hand, and autistic traits and ToM abilities on the other, Pearson correlations were performed. In general, no correlation reached significance. The results are reported in Table 3.

**TABLE 3:** Pearson correlations between the index of the arrow/gaze object dissociation (measured as a difference between the object-cueing effect from arrow and the object-cueing effect from gaze), autistic traits (AQ_Total), and ToM skills (Affective 1; Cognitive 1; Affective 2; Cognitive 2).

	Arrow/Gaze	
	**Object dissociation**	

AQ_Total	Pearson’s r	0.065
	*p*-value	0.647
Affective 1	Pearson’s r	0.258
	*p*-value	0.067
Cognitive 1	Pearson’s r	0.238
	*p*-value	0.092
Affective 2	Pearson’s r	0.003
	*p*-value	0.985
Cognitive 2	Pearson’s r	−0.039
	*p*-value	0.785

## Discussion

Marotta et al. (2012) demonstrated that when arrows were used as cues, attention was spread across the entire spatial extent of objects, whereas when gaze was used, it was selectively oriented toward the specific position or part of the cued object. The present study confirmed this dissociation. Both types of stimuli elicited general cueing effects, while only arrow cues produced object-based effects. No such effect was observed with gaze, which seems to restrict attentional orienting to the part of the object looked at, avoiding the spread of attention across the whole object.

The dissimilarities in the encoding and function of the two types of cue may be the origin of this difference. In particular, Marotta et al. (2012) suggested that a more specific attentional orienting may be triggered by biologically relevant cues. Humans are very accurate in determining where another individual is looking with a direction estimation error ranging from 0.5° to 4° of visual angle (Bock et al., 2008; Wiese et al., 2012). This ability provides important clues for understanding where another person is focusing, helping us to predict their mental states and future actions (Emery, 2000). However, it is not known whether and how the dissociation in attentional selection observed between gaze cues and arrow cues is effectively related to social abilities.

We hypothesized that these differences might be particularly evident in female participants since it has been generally observed that females outperform males in social abilities and cognition. For example, females tend to be more accurate than males at detecting emotional expressions (Suzuki et al., 2006) and to maintain eye contact more frequently and for longer durations (Alwall et al., 2010). However, in the current study, no sex difference was observed in the attentional selection, and the same dissociation between gaze and arrows was observed in female and male participants. These results seem to contradict the intuition that social skills can be associated with the different forms of attentional selection observed between eye-gaze and arrow cues.

However, this conclusion must be interpreted with caution since it was not possible to observe any differences regarding ToM abilities and autistic quotient scores when comparing male and female population in our sample. Thus, this may explain the absence of gender differences observed in the cueing task. It is important to note that most of the participants included in the present study were psychology students. Therefore, although our data are apparently in contrast with studies reporting that females score higher than males on ToM (Kirkland et al., 2013) and lower on the AQ (Baron-Cohen et al., 2001), they are coherent with studies showing that independent of sex, social sciences students have in general greater social skills than students of more “mathematical” sciences (Groen et al., 2018). This would explain the lower scores on the AQ observed in our sample as compared to the general population (13.6 vs. 16.9; see, Ruzich et al., 2015), and the absence of sex differences.

On the other hand, only in female participants were arrows processed faster in comparison to eye-gaze cues. This result is coherent with previous studies and suggests that eye-gaze coding requires some additional time than the coding of arrows (Vlamings et al., 2005; Hietanen et al., 2006; Marotta et al., 2018). It should be noted that schematic faces differ from no-social stimuli such as arrows not just in terms of social significance but also in their complexity. Therefore, it could be argued that a possible explanation for the increase of reaction times for eye-gaze stimuli may reflect their perceptual complexity. However, for the first time, in the present study, we showed that this result could not be extended to the male population since male participants showed no differences in overall reaction time between the two types of stimuli. Therefore, this is more coherent with the “extreme male brain” hypothesis of autism (Baron-Cohen, 2002) according to which male information-processing system is less well adapted for the interpretation and processing of social stimuli than is the female brain. In support of this view, Vlamings and coworkers showed that RTs are slower after eye-gaze than after arrow stimuli in typically developed individuals, but not in individuals with autism. However, we only observed the interaction with sex in the analysis of the general cueing effect. Furthermore, as mentioned above, no sex differences in AQ scores were observed in the present study. Therefore, further research is undoubtedly needed to shed light on this issue. Finally, we tested the hypothesis that the differences between eye-gaze and arrow cues on attentional selection might be related to the individual differences observed on AQ or ToM scores. However, the correlations between gaze-arrow dissociation and both the overall AQ and the ToM scores were non-significant. The fact that the majority of our participants scored low on AQ might at least in part account for these results. Previous studies do not yield a consistent pattern of correlation between AQ and social attention. While some studies suggest a negative correlation between AQ score and gaze-cueing effect (Bayliss et al., 2005; Lassalle and Itier, 2015), another study shows no correlation (Zhao et al., 2015). Further studies will be necessary to shed light on this issue.

## Conclusion

The present study is the first to examine sex differences in attentional object selection triggered by gaze and arrows. The results confirm the existence of distinct modes of attentional selection between these two types of stimuli; in fact, consistent with a previous study (Marotta et al., 2012), both types of stimuli elicited general cueing effects, while only arrow cues produced object-based effects, gaze restricting attentional orienting to the part of the object looked at. However, these differences were not unique to female participants, as no sex differences were observed on attentional effects. Finally, regarding the question of whether the dissociation between gaze and arrows related to social mechanisms, our conclusions are limited, and new research are surely necessary to shed light on this issue.

## Data Availability Statement

The datasets generated for this study are available on request to the corresponding author.

## Ethics Statement

This study was performed in accordance to the ethical standards of Declaration of Helsinki and was approved by the Ethical Committee of the University of Granada (175/CEIH/2017). All participants provided their written informed consent to participate in this study.

## Author Contributions

All authors listed have made a substantial, direct and intellectual contribution to the work, and approved it for publication.

## Conflict of Interest

The authors declare that the research was conducted in the absence of any commercial or financial relationships that could be construed as a potential conflict of interest.

## Footnotes

1 osf.io/tvmk2

2 Research on whether gaze direction and facial expression interact in orienting attention has provided mixed results. While some studies have shown that gaze-cueing effects are independent of facial expression (Hietanen and Leppänen, 2003; Graham et al., 2010; Galfano et al., 2011), others have reported greater gaze-cueing effects when faces show emotional expressions, such as fear or happiness expression (Hori et al., 2005; Bayless et al., 2011; Galfano et al., 2011). However, the impact of the different facial expression have never compared between male and female participants. Further research will be necessary to shed light upon this issue.

3 https://www.autismresearchcentre.com/arc_tests

4 Although it is not germane to the questions addressed in this article, the effect of SOA on cueing effects may be of interest to some readers, and for this reason, it was examined first. Neither the interaction SOA × CT relation (*F* < 1), nor the SOA × Cue Type × CT relation interaction (*p* = 0.335) was significant. The remaining analyses were therefore collapsed across this factor.
